# Increased importance of digital medicine and eHealth during the Covid-19 pandemic

**DOI:** 10.1080/02813432.2020.1770466

**Published:** 2020-06-02

**Authors:** Hans Thulesius

**Affiliations:** Swedish National Editor, Editorial Board Scandinavian Journal of Primary Health Care, Adjunct and Associate Professor of General Practice, Linnaeus University Kalmar and Lund University, Sweden

Digital medicine (also called telemedicine or telehealth) and eHealth form a growing contemporary field in primary health care hastened by the Covid-19 pandemic. The topic is also the theme of the 90th European General Practice Research Network (EGPRN) [1] conference in Gothenburg, which is postponed until May 2021 because of the pandemic.

According to an EU-report 96% of GPs in Europe used electronic health records in 2018 [2]. The Nordic countries together with Estonia, Spain and the UK have adopted eHealth to a high degree and most patients in Denmark, Estonia, Finland, Sweden and the UK can view their medical records and test results electronically [2].

The two highest ranked Swedish primary care dissertations in 2019 (as judged by the Swedish Association of General Practice, SFAM) comprised eHealth approaches and both included randomised control trials. Holst analysed a computerised self-help program for treating depression in her thesis [3]. Nyström et al. assessed an online and a smartphone application for self-management of stress urinary incontinence [4,5].

The subject of digital medicine, telemedicine, eHealth or the use of internet resources was mentioned in the title of 7 out of 104 original articles in the two latest volumes of the Scandinavian Journal of Primary Health Care [5–11].

One of the earliest uses of artificial intelligence in medicine was the efficiency of computer algoritm-based interpretations of ECG to diagnose atrial flutter and atrial fibrillation but a recent study showed that almost 10% of patients in a Swedish primary care context were misclassified by the algorithms [6].

The other six studies involved both patient and physician aspects with four studies emphasising e-learning [5,7,9,10] and two studies using digital data collection methods [8,11].

Our sister journal at Taylor and Francis – the European Journal of General Practice – has published two papers in a series on eHealth in primary care [12,13]. In the first paper it is suggested that eHealth has three main functions:

- to study health parameters (‘inform, monitor and track’)
- to ease communication between healthcare participants (‘interaction’)
- to use health and medical data sources to inform medical decision-making and interventions (‘data utilization’) [14].

The second paper about eHealth from the EJGP deals with the ethical implications of eHealth. This involves issues of predictive and diagnostic uncertainty, roles and responsibilities of patients and physicians, and the patient – physician relationship [13]. Machine-learning and artificial intelligence can indeed offer clinical support but uncertainty cannot be eliminated since algorithms can be invalid or biased as shown in the recent Swedish ECG study mentioned above [6]. Certainly, the roles of patients using eHealth can stimulate self-management and autonomy but also be burdensome causing feelings of isolation. Further, with remote eHealth monitoring of health parameters comes the question ‘who is responsible for monitoring the monitors?’, especially in triage situations. Alas, if face-to-face contact is increasingly replaced by eHealth then the humanitarian quality of primary care may be in jeopardy [13]. This aspect of eHealth was one of the challenges recently discussed by Rudebeck claiming that ‘relationship based general practice may be no more than a historical parenthesis’. [15]. My own reflections as a practicing GP yields a more optimistic view by citing Terentius ‘Homo sum: humani nil a me alienum puto’. (I am human, I consider nothing human alien to me).

Indeed, during the covid-19 pandemic digital contacts have become more common than physical encounters between caregiver and patient [16,17]. The requirements of physical distancing in order to reduce the spread of the virus have in the United States necessitated liberalised legislation for using private communication tools to provide telehealth services during the present public health emergency [18].


Hans Thulesius Swedish National Editor, Editorial Board Scandinavian Journal of Primary Health Care, Adjunct and Associate Professor of General Practice, Linnaeus University Kalmar and Lund University, Sweden hansthulesius@gmail.com

